# The TAFs of TFIID Bind and Rearrange the Topology of the TATA-Less *RPS5* Promoter

**DOI:** 10.3390/ijms20133290

**Published:** 2019-07-04

**Authors:** Sarah N. Le, Christopher R. Brown, Stacy Harvey, Hinrich Boeger, Hans Elmlund, Dominika Elmlund

**Affiliations:** 1 Department of Biochemistry and Molecular Biology, Biomedicine Discovery Institute, Monash University, Clayton, VIC 3800, Australia; 2 ARC Centre of Excellence for Advanced Molecular Imaging, Clayton, VIC 3800, Australia; 3 Department of Molecular, Cell and Developmental Biology, University of California, Santa Cruz, CA 95064, USA; 4 Alnylam Pharmaceuticals, 300 Third St. Cambridge, MA 02142, USA; 5 Two Pore Guys, 2161 Delaware Ave. Suite B, Santa Cruz, CA 95060, USA

**Keywords:** TBP-associated factor, TAF complex, DNA topology, housekeeping gene transcription, ribosomal protein 5

## Abstract

The general transcription factor TFIID is a core promoter selectivity factor that recognizes DNA sequence elements and nucleates the assembly of a pre-initiation complex (PIC). The mechanism by which TFIID recognizes the promoter is poorly understood. The TATA-box binding protein (TBP) is a subunit of the multi-protein TFIID complex believed to be key in this process. We reconstituted transcription from highly purified components on a ribosomal protein gene (*RPS5*) and discovered that TFIIDΔTBP binds and rearranges the promoter DNA topology independent of TBP. TFIIDΔTBP binds ~200 bp of the promoter and changes the DNA topology to a larger extent than the nucleosome core particle. We show that TBP inhibits the DNA binding activities of TFIIDΔTBP and conclude that the complete TFIID complex may represent an auto-inhibited state. Furthermore, we show that the DNA binding activities of TFIIDΔTBP are required for assembly of a PIC poised to select the correct transcription start site (TSS).

## 1. Introduction

About sixty proteins, including RNA polymerase II (Pol II) and the general transcription factors (GTFs), assemble at each promoter prior to each round of transcription [1,2]. This so-called pre-initiation complex (PIC) recognizes the promoter, selects a transcription start site (TSS), and synthesizes a nascent transcript. PIC formation is the key step for regulation of gene expression and represents the end point of many signaling pathways.

Pol II promoters either contain or lack a TATA-box consensus [3]. Genes with the TATA-box promoter are often under stringent regulation to enable a rapid response to cellular stress. However, most (~80%) Pol II promoters do not contain a canonical TATA-box [3,4,5,6]. These TATA-less genes are constitutively transcribed and frequently involved in “housekeeping” processes, essential for all basic cellular maintenance.

The crystal structure of the TATA-box binding protein (TBP) bound to the adenovirus major late TATA promoter revealed a saddle-shaped TBP molecule, stabilizing an ~80° kink of the promoter by binding the partially unwound TATA-box DNA helix [7]. That structure established a view of the TBP-promoter complex as a nucleation point for PIC assembly. However, transcription from Pol II promoters rarely involves the classical TATA-box. TBP is nevertheless essential for transcription of all genes. Analysis of in vivo expression levels in yeast strains with mutations in the DNA binding surface of TBP addressed the question of whether the DNA binding activity of TBP is required for transcription from TATA-less promoters [8]. Most mutants did not support transcription from TATA-containing promoters, whereas TATA-less promoters were largely unaffected. The specific DNA binding activity of TBP appears not to be important for the transcription of “housekeeping” genes with TATA-less promoters. Other components of the general transcription machinery must therefore provide the nucleation point for transcription initiation on TATA-less promoter types.

The general transcription factor TFIID is a mega-Dalton sized complex composed of TBP and 14 TBP-associated factors (TAFs). Previous in vivo studies position TFIID exclusively on TATA-less promoters [6,9,10]. However, recent findings position TFIID on both classes of promoters [11,12]. TFIID binds ~200 base pairs (bp) of DNA upstream and downstream of a cluster of 5–7 TSSs in vivo [10,13]. Despite many decades of TFIID research, there are no DNase I footprints that agree with the extended DNA binding observed in vivo. Published transcription assays are generally supplemented with whole cell extracts or utilize non-native TATA-box promoters [14,15,16,17,18,19].

TBP dynamically associates with the complex of TAFs (TFIIDΔTBP) and as a result purified TFIID has ~50% TBP occupancy [17,20]. No specific functional roles have been attributed to either TAF complexes containing or lacking TBP in vitro. However, it has been reported that recruitment of TAFs to TATA-less promoters does not require TBP in vivo, raising the possibility that TAFs might be constitutively bound to the core promoter during active transcription [6,21].

In this study, we tested the hypothesis that the TAF complex (TFIIDΔTBP) recognizes the TATA-less promoter of the ribosomal protein 5 gene (*RPS5*) independent of TBP in vitro. We found that the TAF complex binds ~200 bp of the promoter and dramatically changes the DNA topology. Unexpectedly, we also found that TBP inhibits the DNA binding activities of the TAF complex. Our data suggest that binding of TBP to the TAFs of TFIID represents a regulatory switch. TFIID may represent an auto-inhibited state, incapable of binding the promoter and rearranging its topology. Instead, it is the TBP-lacking TAF complex that binds the promoter and rearranges its topology, thereby providing the nucleation point for assembly of a PIC poised to select the correct TSS. We propose that the TAFs–DNA rather than the TBP–DNA complex represents the nucleation point for PIC assembly on the TATA-less *RPS5* promoter.

## 2. Results

### 2.1. Purification of TFIID∆TBP (TAF Complex)

**Figure 1:**
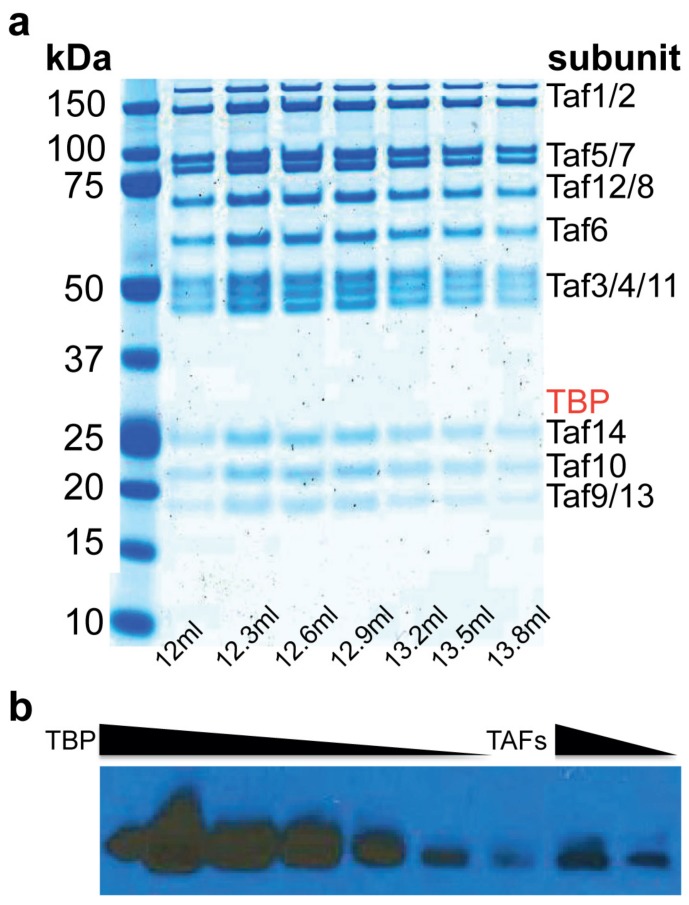
Purification of the TATA-box binding protein (TBP)-associated factor (TAF) complex. (**a**) SDS–PAGE of TAF complex fractions from size-exclusion chromatography. Elution volumes indicated. (**b**) Quantification of TBP in the TAF complex preparation by immunoblotting against known amounts of TBP revealed less than 3% TBP contamination (a two-fold dilution series of recombinant TBP from 2 pmoles to 12.5 fmols and 2 and 1 pmols of purified TAF complex were loaded on the gel).

**Figure 2:**
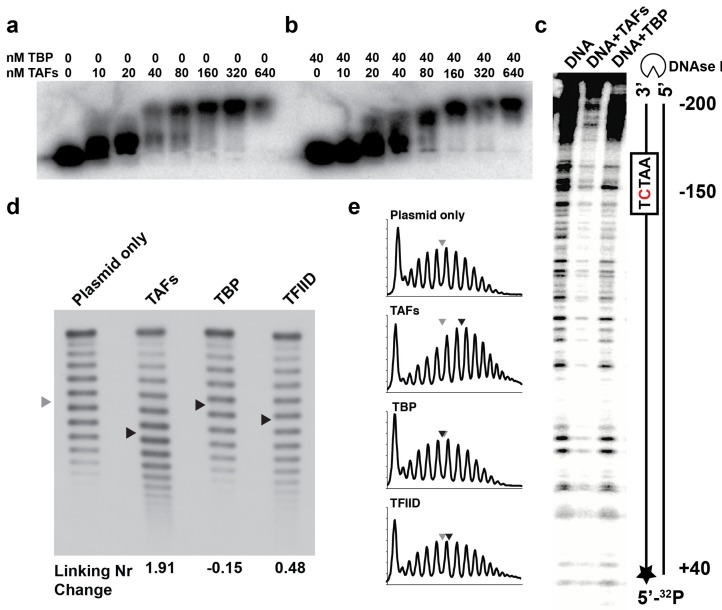
DNA binding experiments. (**a**) Electrophoretic mobility gel-shift assay (EMSA) in 0.8% Mg–agarose gel showed that the TAF complex binds the *RPS5* promoter with nM affinity. (**b**) EMSA with addition of a constant amount of TBP while titrating TAFs. (**c**) DNAse I footprinting assay with 240 bp of *RPS5* promoter. The TAF complex introduced an extended footprint, spanning ~200 bp DNA. TBP introduced a small footprint in the region around the pseudo-TATA-box. (**d**) Topoisomerase I assay showed a fourfold increase in positive supercoiling for TAFs vs TFIID. The centers of the distributions are indicated with arrows. (**e**) Intensity distribution profiles of the bands in (**d**).

A TAF complex fully depleted of TBP has not previously been purified to homogeneity. We surmounted this challenge and developed a purification protocol that yielded the complete *Saccharomyces cerevisiae* TAF complex, containing TAF1–TAF14 in stoichiometric amounts (Figure 1a and Supplementary Figure S1) with less than 3% TBP contamination, as measured by immunoblotting (Figure 1b). The presence of all 14 TAFs was confirmed by mass spectrometry (Supplementary Figure S2). Electrophoretic mobility gel-shift assay (EMSA) revealed that the TAF complex binds the *RPS5* promoter with nM affinity (Figure 2a).

We showed that TBP alone binds *RPS5*, forming at least two specific complexes with one or more TBP molecules bound, which agrees with previously published DNAse I footprinting of TBP on the *RPS5* promoter [8] (Supplementary Figure S3). Pre-incubation of a constant amount of TBP with an increasing amount of TAFs before addition of DNA in our EMSA shows that TBP has an inhibitory effect on the DNA binding activity of the TAFs. A slight shift was observed with TAFs and TBP present in equimolar amounts (i.e., the complete TFIID complex) (Figure 2b). There was no complete shift at 20 nM of TAF in the presence of 40 nM of TBP, but rather a smear of the free DNA label, which we interpret as free TBP binding to the probe (see Supplementary Figure S3). Our EMSA results showed that TBP inhibits TAFs–DNA interactions. Only in the presence of saturating amounts of TAFs vs TBP was a clear shift observed (Figure 2b).

### 2.2. The TAFs Alone Bind and Introduce Significant Topological Changes to the TATA-Less RPS5 Promoter

To further characterize the TAFs–DNA interactions, we measured the coverage of DNA by the TAF complex using a DNase I footprinting assay with 240 bp of *RPS5* (160 bp upstream and 80 bp downstream of the TSS). We observed an extended footprint, spanning almost the entire length of the promoter (Figure 2c). Binding of the TAFs to DNA both upstream and downstream of the TSS was consistent with previous in vivo studies [10,13]. We concluded that the TAF complex alone binds a large part (~200 bp) of the *RPS5* promoter independent of TBP.

Finally, we integrated *RPS5* into a plasmid and measured the DNA topological changes introduced by the TAF complex, TBP, and TFIID, respectively, using a topoisomerase I assay (Figure 2d,e; the centers of the distributions are indicated with arrows). Protein binding to a supercoiled plasmid affects DNA topology by changing the local DNA twist, which destabilizes the duplex and generates denatured regions of DNA. In our experiments, analysis of the topoisomer distribution for the TAF complex revealed a large linking number change (ΔLk) of +1.91. Thus, the TAFs alone change the promoter DNA topology to a larger extent than the nucleosome core particle, which introduces a ΔLk of −1 to −1.25 [22]. The opposite sign of the ΔLk suggests that the TAF complex wraps DNA in the opposite direction to the nucleosome or increases the twist of the DNA. TBP introduced a small negative ΔLk, consistent with previous reports [6], and local untwisting of DNA by TBP. Incubating the plasmid with TFIID (TAFs and TBP in equimolar amounts mixed before the addition of plasmid DNA) reduced ΔLk to +0.48. The similarity between TBP and TFIID topoisomer distribution is consistent with our other results, which show that TBP interferes with TAFs binding to the promoter. In the topoisomerase experiment, we added saturating amounts of proteins vs plasmid. In the TFIID sample there are three different species (TBP, TAFs, and TAFs–TBP), which compete for the promoter DNA. The wider distribution in the TFIID sample can therefore be explained by greater conformational and structural heterogeneity. The much wider topoisomer distribution of the TAFs sample vs TBP and TFIID shows that TAFs alone can bind and rearrange the RPS5 promoter topology without TBP, and addition of TBP affects this behavior. The strong band at the top of the gel corresponds to the nicked plasmid.

### 2.3. The TAF Complex Provides the Nucleation Point for Correct Housekeeping Gene Transcription In Vitro

In vitro run-off transcription from the *HIS4* TATA promoter produces mRNAs of length consistent with a TSS located 25 bp downstream of the TATA-box [19,20] (Supplementary Figure S4). On TATA-less promoters, the distance between the pseudo-TATA-box and the cluster of 5–7 in vivo TSSs [13] is typically much longer and more variable (~80 bp on *RPS5*; Supplementary Figure S5).

**Figure 3:**
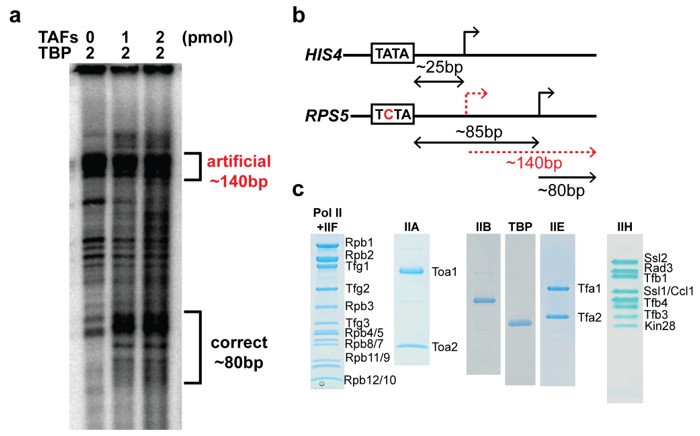
Reconstitution of transcription from the housekeeping *RPS5* gene. (**a**) Run-off transcription gel. (**b**) Schematic representation of the TATA-containing *HIS4* and the TATA-less *RPS5* promoters. The artificial TSS due to TBP-dependent not TAF-dependent transcription is indicated with a dashed red arrow. The length of the artificial and correct transcripts on the *RPS5* promoter are indicated with a red dashed and black arrow below the *RPS5* promoter schematic picture. (**c**) Purification of general transcription factors (GTFs). SDS–PAGE of the remaining purified GTFs with the individual subunits indicated for multi-subunit GTFs.

We reconstituted the housekeeping transcription reaction from highly purified components (Figure 3c). First, we established that the purified factors (omitting the TAFs) were active by performing the run-off transcription assay on the *HIS4* TATA promoter (Supplementary Figure S4; lane 1), as described previously [23,24]. Next, we showed that TBP alone, in the absence of TAFs, was capable of nucleating a PIC and initiating transcription from a non-native TSS on *RPS5* (Figure 3a; lane 3 and Supplementary Figure S4; lane 3). In both assays, a strong band was observed corresponding to a non-native transcript of around 140 bp, which was located approximately 25 bp downstream from the pseudo-TATA box. The characteristic cluster of specific *RPS5* mRNA products [9] was detected only with TAFs present in the reaction (Figure 3a; lanes 2,3). The presence of a strong TBP-dependent artificial mRNA product in all reactions suggests that a population of TAF-containing and TAF-lacking PICs was formed in vitro. The TAF-lacking PIC was presumably formed through an assembly pathway similar to that characterized for the *HIS4* TATA promoter [23,24].

### 2.4. Structural Rearrangements of DNA Bound and Unbound TAF Complexes

**Figure 4:**
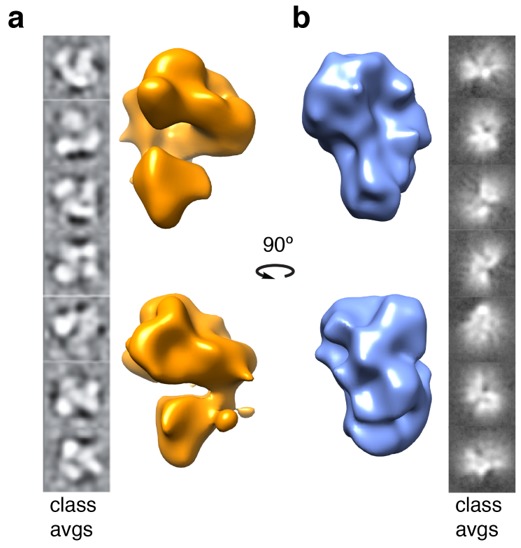
Preliminary 3D reconstructions and corresponding class averages of apo-TAFs and TAFs–DNA complexes. (**a**) apo-TAF complex (orange) showing the distinct horseshoe-shaped 3-lobed structure. (**b**) TAFs in complex with ~200 bp of *RPS5* promoter (blue) showing a globular nucleosome-like core structure with extra density around the entire molecule, which we attribute to the wrapping of DNA by the TAFs. These preliminarily 3D reconstructions indicate a visually distinct difference between the DNA bound and unbound TAF complexes.

We sought to solve the cryo-electron microscopy (EM) structures of apo-TAFs and TAFs–DNA, to understand at the molecular level where the TAFs contact promoter DNA and what topological changes are introduced. Three-dimensional reconstruction of both apo-TAFs and TAFs–DNA complexes stalled at ~20 Å. We concluded that further stabilization of both complexes was required as the inherent heterogeneity present in both samples posed a real challenge for further high-resolution studies. Preliminary 3D reconstructions indicated visually distinct differences between the DNA bound and unbound TAF complexes. The apo-TAF complex showed the expected horseshoe-shaped 3-lobed structure, consistent with previous studies (Figure 4a) [25]. In contrast, when the TAFs were bound to ~200 bp of promoter DNA, the molecule appeared to adopt a more globular nucleosome-like structure, with the DNA path wrapping the entire molecule, presumably to bring the TSS closer to the pseudo-TATA-box (Figure 4b).

We did not observe protruding DNA ends, neither in class averages nor in the 3D reconstructions. The apo structure was 220 Å in the longest direction and the DNA bound structure was compacted to 180 Å. The DNA used in the EM studies was ~200 bp, which corresponded to 680 Å. The extended footprint of around 200 bp (Figure 2c) suggested that the DNA was wrapped around the TAFs. Furthermore, the large linking number change (ΔLk) of +1.91 introduced by the TAFs, which was almost double that of the nucleosome, suggested extensive DNA wrapping. Visualization of the mobility of the different lobes and how this mobility was coordinated to orchestrate the conformational changes required to accomplish transcription initiation by the TAFs of TFIID remains a challenge.

## 3. Discussion

We show that the TAFs of TFIID recognize the *RPS5* promoter and rearrange its topology independent of TBP. Our data supports the idea that the TAFs of TFIID act as core promoter selectivity factors independent of TBP in vivo [6]. Unexpectedly, we find that TBP interferes with the DNA binding activity of the TAF complex. When TBP is pre-incubated with TAFs in equimolar amounts (i.e., TFIID) before addition of DNA in the gel shift assay, the bands become smeary and no clear shift is observed. The TAF complex alone, in contrast, produces a clear DNA shift (Figure 2a,b; compare bracketed lanes). Furthermore, the TAF activities responsible for the dramatic change in DNA topology are inhibited in the presence of TBP (Figure 2d; compare lanes “TAFs” and “TFIID”).

**Figure 5:**
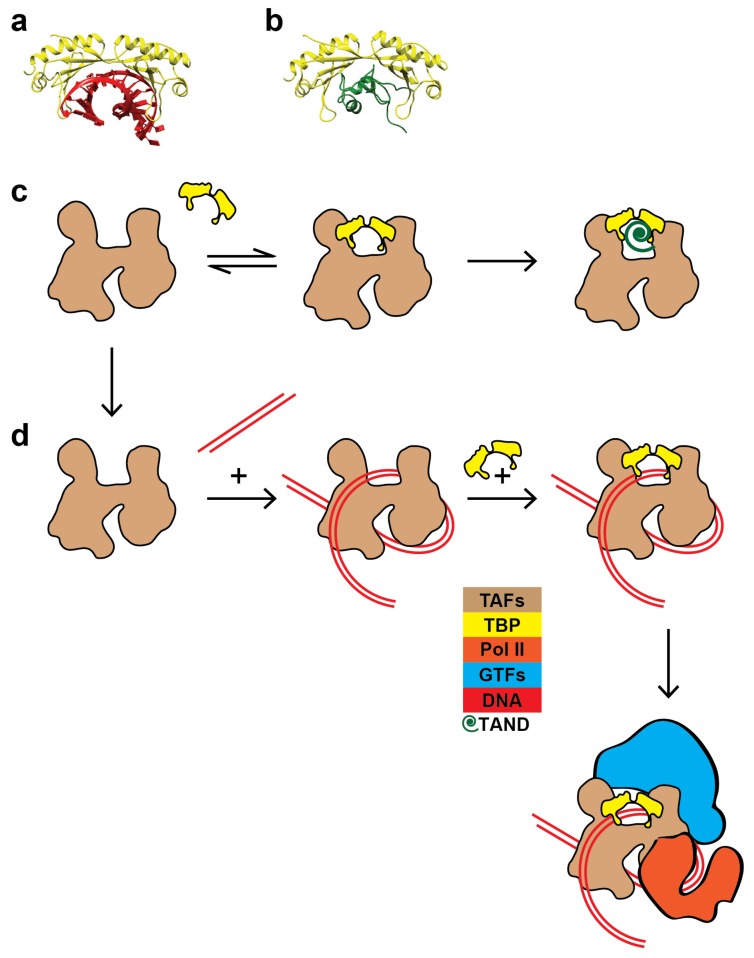
Schematic of TAFs–DNA assembly pathways in vitro. (**a**) X-ray crystal structure of TBP in complex with TATA-box DNA (PDB:1YTB). (**b**) NMR structure of TBP in complex with the N-terminal domain of TAF1 (TAND) (PDB:1TBA). (**c**) Dynamic association of TBP with its TAFs. When TBP is associated with its TAFs, TBP engages with the TAND domain of TAF1 and represents an auto-inhibited TFIID complex unable to bind and recognize promoter DNA. (**d**) The pre-initiation complex (PIC) assembly pathway that we propose for the TATA-less promoter of the *RPS5* gene. When the TAFs are not bound to TBP, or once the TBP–TAFs interaction is broken, DNA binds the TAFs and TBP is then engaged at the promoter on lobe A of TFIID. The TAF complex alone rearranges the DNA topology, providing the nucleation point required for assembly of a PIC poised to select the correct TSS.

There are structural data that relate to our findings. *Schizosaccharomyces pombe* TAFs–DNA, TAF complex, and TFIID cryo-EM reconstructions showed that TBP, when bound to the TAFs, places a molecular lid over the DNA binding furrow of the TAF complex [20]. Furthermore, the NMR structure of the N-terminal domain of TAF1 (TAND) in complex with TBP [26] showed that TAND occupies the very same position as the DNA would in the TBP–DNA crystal structure [7] (Figure 5a,b). The inescapable conclusion is that TBP has two mutually exclusive binding partners within the TAF complex. TBP either interacts with a promoter segment pre-bound to TAFs or, when TAFs are not engaged with DNA, TBP interacts with the TAND domain of TAF1.

Our data suggest that binding of TBP to the TAFs of TFIID represents a regulatory switch. The TFIID molecule represents an auto-inhibited state, incapable of binding and rearranging the promoter DNA topology. A plausible explanation for this phenomenon is that TBP, when bound to the TAFs, inhibits TAFs–DNA interactions [20] (Figure 2b,d,e). Previous studies show that TFIID binds DNA only when TFIIA is present to reverse this inhibition [16]. Furthermore, quantitative analysis of in vivo transcription levels has shown that many activators fused to DNA-binding domains work better in cells with TAND deletions [27]. In light of our findings, that deletion of parts of TAND may decrease the affinity of TBP for TAFs and shift the equilibrium between the TAFs and the auto-inhibited TFIID molecule toward the DNA-binding TAF complex.

All currently available structural data of TFIID in complex with the promoter have been obtained with short DNA sequences. Recent cryo-EM TFIID structures with a promoter segment of 8 bp indicate no wrapping of DNA by the TAFs [25], whereas our preliminary cryo-EM 3D reconstructions show a more compact globular structure when TAFs are bound to ~200 bp of DNA, presumably due to DNA wrapping (Figure 4b). Taken together, our data show that by using a 240 bp TATA-less *RPS5* promoter, the TAFs of TFIID bind and wrap DNA in the opposite direction to the nucleosome core particle.

Our study indicates a role of TAFs beyond simply recognizing promoter DNA. TAFs are required for the assembly of a PIC poised to select the correct TSS on the TATA-less *RPS5* promoter. That such an extended footprint and large change to DNA topology is correlated with correct TSS selection is an unexpected finding. Perhaps this substantial change to DNA topology is a hallmark of housekeeping gene transcription.

TBP or other GTFs may bind the TATA-less promoter before TAFs in vivo, followed by recruitment of the TAFs and the remaining GTFs. However, this pathway is unlikely to result in the formation of a PIC poised to select the correct TSS in vitro, since a mixture of TAF-dependent and TBP-independent PICs are formed (Figure 3a). Instead, we conclude that TBP and the other GTFs direct the PIC assembly on *RPS5* after the formation of a TAFs–DNA complex (Figure 5c,d). Our findings agree with the recently published human TFIID structure [25], where the authors observe that TAF11 and TAF13 interact with TBP and keep TBP inhibited before the TAFs are bound to the promoter.

We discovered that the TAFs of TFIID introduce a significantly rearranged *RPS5* promoter topology, required for correct start-site selection in vitro. Our study lays the foundation for further mechanistic and structural studies of housekeeping transcription, involving co-regulators, activators, and repressors.

## 4. Materials and Methods

### 4.1. Oligonucleotides

A 240 bp *RPS5* (−180/+60) DNA fragment was amplified by PCR from yeast genomic DNA using primers with EcoRV sites. The fragment was subsequently TA-cloned into a *pDrive* vector. The plasmid construct was amplified in *E. coli* and purified and digested with EcoRV. The promoter fragment was agarose gel purified, ethanol precipitated, and re-suspended in water. Non-native ATC and GAT were retained at the 5′- and 3′-ends.

### 4.2. Protein Purification

The TAF complex was purified from a *Saccharomyces cerevisiae* TAF1–TAP tagged strain. A 36 L yeast culture was grown to OD = 6–7 and cells harvested by centrifugation. Harvested cells were resuspended in equal volume lysis buffer (100 mM HEPES pH 7.8, 400 mM potassium acetate, 4 mM magnesium sulfate, 10% glycerol, 10 mM DTT, and protease inhibitor cocktail: PMSF 3.4% (w/v), leupeptin 5.68% (w/v), pepstatin A 0.0274% (w/v), and benzamidine HCl 6.6% (w/v)), and lysed in a DynoMill (Multi Lab WAB, Blaxland, Australia). Cellular debris was removed by centrifugation in a JLA8.1 rotor (Beckman Coulter, Sydney, Australia) at 8000× rpm, 4 °C for 1 h, and the supernatant was adjusted to 600 mM potassium acetate. To remove nucleic acids, PEI (50% w/v) was added to the lysate to a final concentration of 0.1%. The lysate was further clarified by ultracentrifugation in a Ti-45 rotor (Beckman Coulter) at 45,000× rpm for 1 h. The cleared supernatant was loaded onto an IgG column (10 mL of 10 mg/mL IgG resin). Bound complex was washed with 1 L of high salt buffer (50 mM HEPES pH 7.8, 1 M potassium acetate, 2 mM magnesium sulfate, 5% glycerol, 1 mM DTT, and protease inhibitor cocktail), 500 mL of Low Salt Buffer (50 mM HEPES pH 7.8, 50 mM potassium acetate, 2 mM magnesium sulfate, 5% glycerol, 1 mM DTT, and protease inhibitors) and equilibrated in 200 mL TEV protease Buffer (50 mM HEPES pH 7.8, 200 mM potassium acetate, 2 mM magnesium sulfate, 5% glycerol, and 1 mM DTT). The bound complex was cleaved overnight with 100 µg TEV protease at 4 °C and eluted in 5 column volumes of gel filtration buffer (50 mM HEPES pH 7.8, 400 mM potassium acetate, 4 mM magnesium sulfate, and 1 mM DTT), concentrated and loaded onto a pre-equilibrated Superose 6 10/300 column (GE Healthcare, Melbourne, Australia). The TAF-complex eluted as a single symmetric peak centered at around 12.6 mL. The other GTFs and Pol II were purified as previously described [17,19,20].

### 4.3. Quantitation of the Amount of TBP in the TAF Complex Preparation

Quantitation of the amount of TBP was done by immunoblotting. A two-fold dilution series of recombinant TBP (2 pmoles to 12.5 fmols) and 2 and 1 pmols of purified TAF complex from above were loaded on SDS–PAGE and electrophoresed. The proteins were then transferred to a PVDF membrane using a western transfer system (Bio-Rad, Gladesville, Australia). The blot was blocked with 5% skim milk in TBST, followed by incubation with anti-TBP antibody (Abcam, cat# ab28175) and secondary antibody (Abcam, San Francisco, CA, USA), washed 3 times with TBST, and developed. The blot was scanned, and the intensities of the bands were quantitated using ImageJ (Version 1.51, NIH).

### 4.4. Electrophoretic Mobility Shift Assay

*RPS5* promoter was end-labeled with (γ-^32^P)ATP using T4 polynucleotide kinase. Ten μL binding reactions contained a serial dilution of TAFs, or a serial dilution of TAFs preincubated with a constant amount of TBP, in protein binding buffer (20 mM HEPES, 100 mM potassium acetate, 2 mM magnesium acetate, 2 mM DTT, 10 μg of BSA, and 5% glycerol), and a 10,000 cpm probe (32 fmoles). An unrelated competitor DNA of the same length was added in 100 fold excess to prevent unspecific binding. After 30 min of incubation at room temperature, reactions were resolved on a 0.8% agarose gel in 45 mM boric acid, 5 mM magnesium acetate, and 45 mM Tris. The 4 mm thick gels were run at 7.5 V/cm for 50 min at 4 °C. Gels were dried, exposed overnight, and quantitation was performed with the use of a PhosphorImager (GE Healthcare, Melbourne, Australia)and ImageQuant software (TL 7.0, GE Healthcare, Sweden). The TBP/*RPS5* gel shift was performed as described above, and reactions were resolved on a 2% agarose gel.

### 4.5. DNase I Footprinting

The *RPS5* promoter was end-labeled with (γ-^32^P)ATP. Five fmol *RPS5* was incubated for 20 min at 30 °C in protein binding buffer (25 mM HEPES pH 7.8, 150 mM potassium acetate, 3 mM magnesium acetate, 7% glycerol, and 2 mM DTT) alone or with 2 pmol TAFs or TBP. DNase I (0.1 U) was added for 1 min and reactions were terminated with stop buffer (400 mM sodium acetate, 0.2% SDS, and 10 mM EDTA). Samples were digested with proteinase K, phenol/chloroform extracted, and electrophoresed on an 8% polyacrylamide/urea gel at 10 W for 2.5 h. Gels were dried, exposed overnight, and quantitation performed using a PhosphorImager and the ImageQuant software.

### 4.6. Topological Analysis

*pDrive/RPS5* was incubated with TAFs, TBP, and TAFs and TBP (TFIID), respectively, in protein binding buffer (25 mM HEPES pH 7.8, 150 mM potassium acetate, 3 mM magnesium acetate, 7% glycerol, 2 mM DTT) for 20 min at 30 °C. Topoisomerase I (1 U) was added and samples were incubated at 37 °C for 1 h, followed by addition of 0.5% SDS and digestion with proteinase K. DNA samples were purified with a Zymo DNA clean/concentrator Kit. Analysis of topoisomer distributions was performed as previously described [28], except DNA was electrophoresed in a 0.9% agarose gel containing 8 µM chloroquine at 1.2 V/cm for 42 hr, blotted, and hybridized with radiolabeled DNA probe (against *RPS5*) as previously described [24].

### 4.7. Run-Off Transcription Assay

One pmol of *RPS5* template was mixed with 0, 1, and 2 pmol of TAFs, in 2 μL of transcription buffer (50 mM HEPES pH 7.6, 300 mM potassium acetate, 5 mM magnesium sulfate, 5% glycerol, and 5 mM DTT) and incubated for 15 min on ice, followed by addition of 2 pmol of TBP and further incubation for 15 min. Then, 2 pmols of TFIIA, -B, -E, and -H were added to the reaction mixture and the potassium acetate concentration was adjusted to 90 mM followed by 15 min incubation on ice. Finally, 1 pmol Pol II-TFIIF complex was added and potassium acetate concentration was further diluted to 30 mM and magnesium sulfate adjusted to 7.5 mM in 20 μL total reaction volume. Transcription was initiated by adding an equal volume of 2X transcription mixture (1.6 mM ATP, 1.6 mM GTP, 1.6 mM CTP, 40 μM UTP, 0.083 μM (α-^32^P)UTP (2.5 μCi), 10 mM magnesium acetate, and 5 units of RNaseOUT) at 30 °C. The reaction was stopped after 20 min by addition of 185 μL of stop buffer (10 mM Tris pH 7.5, 300 mM sodium acetate pH 5.5, 5 mM EDTA, 0.7% SDS, 0.1 mg/mL glycogen, and 0.013 mg/mL proteinase K). mRNA products were ethanol precipitated, resuspended in loading buffer, and resolved on a denaturing 6% polyacrylamide/urea gel. Gels were exposed overnight and quantitation was performed using a PhosphorImager and the ImageQuant software. Control reactions using *HIS4* (−96/+112) were performed as described above, but without the addition of TAFs.

### 4.8. 3D Reconstructions of Apo-TAFs and TAFs–DNA Complexes

For cryo-EM sample preparation, purified TAF complex was stabilized using the GraFix method [29,30] and DNA was added in 2x molar excess and incubated for 15 min at room temperature for the TAFs–DNA complex. Then, 4 µL of each protein sample was applied to a Quantifoil R1.2/1.3 holey carbon grid and rapidly frozen in liquid ethane using a Vitrobot IV (FEI). The sample-containing grid was then loaded into the Titan Krios FEG–TEM 300 kV instrument equipped with a K2 direct electron detector (Gatan) and imaged under low-dose conditions (10–15 e/Å^2^). Collected movies were motion and CTF corrected using SIMPLE [31,32], and particles were manually picked using EMAN2 [33]. Data processing was performed using SIMPLE [31,32], and depictions of 3D reconstructed models were generated using UCSF Chimera [34].

## Supplementary Materials

Supplementary materials can be found at https://www.mdpi.com/1422-0067/20/13/3290/s1.

## Abbreviations

bp	Base pairs
EMSA	Electrophoretic mobility gel-shift assay
GTFs	General transcription factors
ΔLk	Linking number change
PIC	Pre-initiation complex
Pol II	RNA polymerase II
RPS5	Ribosomal protein 5
TAFs	TBP-associated factors
TAND	N-terminal domain of TAF1
TBP	TATA-box binding protein
TSS	Transcription start site
