# Supplementary material for: The TAFs of TFIID Bind and Rearrange the Topology of the TATA-Less RPS5 Promoter
Source: Int J Mol Sci. 2019 Jul 4;20(13):3290. doi: 10.3390/ijms20133290 (PMC6650902; doi:10.3390/ijms20133290)
Supplement: Supplementary file 1 [file ijms-20-03290-s001.pdf]

SUPPLEMENTARY MATERIALS accompanying the manuscript "The TAFs of TFIID bind and rearrange the topology of the TATA-less *RPS5* promoter"

SUPPLEMENTARY FIGURE LEGENDS

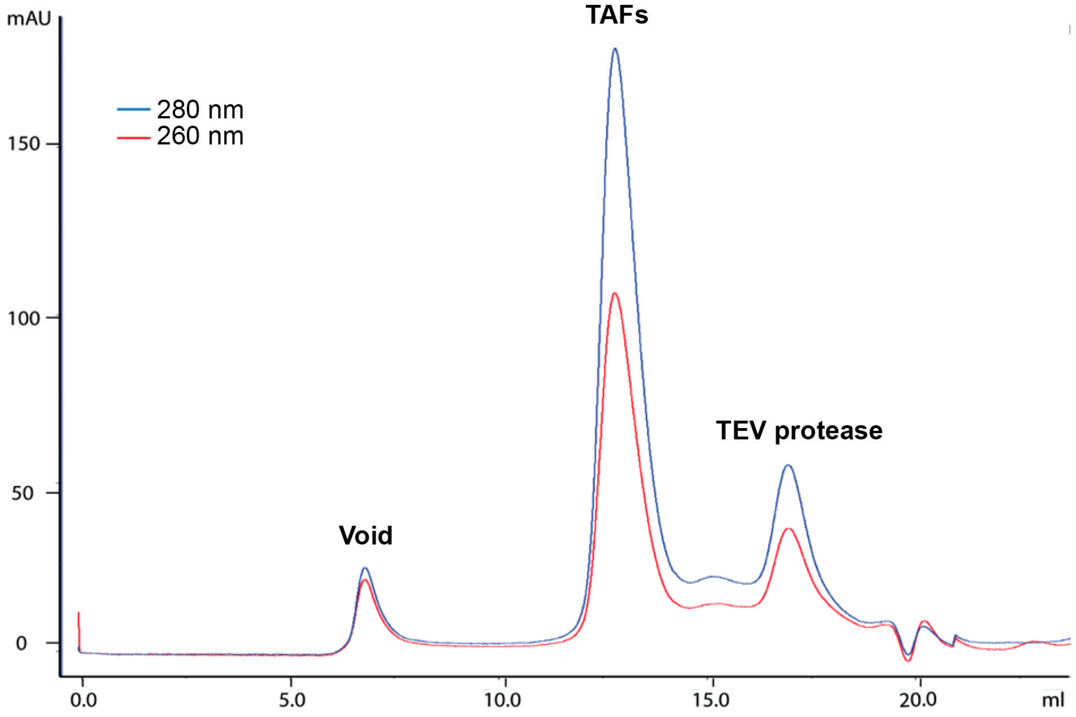

Supplementary Figure S1. A size-exclusion chromatography profile of the TAF complex purification.

TAF5_YEAST **Mass:** 89426 **Score:** 1049 **Matches:** 30(26) **Sequences:** 25(23) **emPAI:** 1.74
Transcription initiation factor TFIID subunit 5 OS=Saccharomyces cerevisiae (strain ATCC 204508 / S288c) GN=TAF5 PE=1 SV=1
☐ Check to include this hit in error tolerant search or archive report

	Query	Observed	Mr(expt)	Mr(calc)	ppm	Miss	Score	Expect	Rank	Unique	Peptide
☑	50	496.2864	990.5582	990.5750	-16.88	0	47	0.031	1	U	R.IVLEYLNK.K
☑	55	506.2456	1010.4766	1010.4920	-15.29	0	52	0.0054	1	U	K.YLLSGSEDK.T
☑	73	537.7444	1073.4742	1073.4890	-13.79	0	43	0.016	1	U	K.DFHGSEINR.L
☑	92	573.2864	1144.5582	1144.5764	-15.89	0	61	0.00082	1	U	K.NAIYSLSYSK.E
☑	110	587.3151	1172.6156	1172.6302	-12.45	0	50	0.017	1	U	R.SNLALAGGAFRP.-
☑	139	626.7771	1251.5396	1251.5554	-12.65	0	68	4.7e-005	1	U	R.MWDVSTGDSVR.L
☑	146	632.7916	1263.5687	1263.5844	-12.39	0	43	0.027	1	U	R.SNGPFSASDLNR.I
☑	162	642.3332	1282.6518	1282.6557	-3.06	0	72	7e-005	1	U	R.IAAAGFQDSYIK.I
☑	189	673.8178	1345.6211	1345.6262	-3.83	0	63	0.00029	1	U	K.ENEVASAFQSHK.Y
☑	208	694.8590	1387.7034	1387.7095	-4.41	0	74	5.1e-005	1	U	R.LEGLNAPENYIR.A
☑	244	729.3942	1456.7738	1456.7885	-10.13	0	68	0.00019	1	U	K.TAKPISNPTNLSSK.R
☑	272	762.8820	1523.7494	1523.7692	-13.01	0	55	0.0035	1	U	K.EGNVLISGGADHTVR.V
☑	304	821.4386	1640.8627	1640.8662	-2.10	0	92	7.3e-007	1	U	R.TVIPTSDLVASFYTK.K
☑	366	925.9168	1849.8191	1849.8303	-6.02	0	81	2.7e-006	1	U	K.QLNQQTAGDNYSGANNR.T
☑	380	635.9650	1904.8732	1904.9089	-18.76	0	34	0.19	1	U	K.QSTNQNQNGTHQPQPVK.N
☑	407	998.9779	1995.9412	1995.9538	-6.33	0	(47)	0.014	1	U	K.TLVGHSGTVYSTSFSPDNK.Y
☑	408	666.3210	1995.9412	1995.9538	-6.33	0	66	0.00015	1	U	K.TLVGHSGTVYSTSFSPDNK.Y
☑	414	1012.0349	2022.0552	2022.0721	-8.33	0	(32)	0.48	1	U	R.LFLGHTAPVISIAVCPDGR.W
☑	415	675.0257	2022.0554	2022.0721	-8.27	0	46	0.02	1	U	R.LFLGHTAPVISIAVCPDGR.W
☑	448	1074.5279	2147.0413	2147.0535	-5.68	0	(11)	66	1	U	R.WLSTGSEDGIINVWDIGTGK.R
☑	449	1074.5291	2147.0436	2147.0535	-4.60	0	119	1e-009	1	U	R.WLSTGSEDGIINVWDIGTGK.R
☑	450	716.6885	2147.0436	2147.0535	-4.60	0	(65)	0.00026	1	U	R.WLSTGSEDGIINVWDIGTGK.R
☑	580	900.0725	2697.1958	2697.2200	-8.96	0	41	0.017	1	U	R.TNNAAGANSGQQPQQQSQGQSQQQGR.S
☑	626	1026.8220	3077.4442	3077.4717	-8.93	0	62	0.00022	1	U	K.ATTEPSAEPDEPFIGYLGDVTASINQDIK.E
☑	629	627.6931	3133.4294	3133.4543	-7.97	0	(106)	5.4e-009	1	U	K.GHNHPVWDVSFSPLGHYFATASHDQTAR.L
☑	630	784.3650	3133.4309	3133.4543	-7.48	0	112	1.6e-009	1	U	K.GHNHPVWDVSFSPLGHYFATASHDQTAR.L
☑	633	1048.8174	3143.4302	3143.4465	-5.19	1	80	2.2e-006	1	U	K.IWSLDGSSLNNPNIALNNNDKDEDPTCK.T
☑	641	1069.5186	3205.5341	3205.5667	-10.17	1	65	0.00013	1	U	K.KATTEPSAEPDEPFIGYLGDVTASINQDIK.E
☑	680	971.2566	3880.9973	3881.0405	-11.14	0	54	0.00084	1	U	K.NWVDSSLEIYKPELSYIMYPIFIYLFLNLVAK.N
☑	708	1146.5968	4582.3582	4581.4061	208	1	1	1e+002	1	U	K.NWVDSSLEIYKPELSYIMYPIFIYLFLNLVAKNPVYAR.R

TAF2_YEAST **Mass:** 162851 **Score:** 701 **Matches:** 26(17) **Sequences:** 24(16) **emPAI:** 0.52
Transcription initiation factor TFIID subunit 2 OS=Saccharomyces cerevisiae (strain ATCC 204508 / S288c) GN=TAF2 PE=1 SV=3
☐ Check to include this hit in error tolerant search or archive report

	Query	Observed	Mr(expt)	Mr(calc)	ppm	Miss	Score	Expect	Rank	Unique	Peptide
☑	41	481.7412	961.4679	961.4902	-23.22	0	48	0.021	1	U	R.LAACEALSK.Y
☑	56	506.2532	1010.4918	1010.5073	-15.36	0	49	0.014	1	U	K.VVGEEGFFK.S
☑	59	510.7541	1019.4936	1019.5135	-19.47	0	27	2.7	1	U	R.TSESNELLK.Q
☑	104	581.8168	1161.6191	1161.6394	-17.45	0	35	0.48	1	U	K.ELPHPIDLTK.K
☑	119	601.8460	1201.6774	1201.6958	-15.29	0	36	0.35	1	U	R.LLYPLEVIDK.A
☑	124	607.2935	1212.5725	1212.6099	-30.81	0	63	0.00037	1	U	K.SSVQSVHNEVK.N
☑	150	636.8050	1271.5954	1271.6106	-11.91	0	60	0.00069	1	U	R.NIEESNNPSLR.D
☑	169	648.3343	1294.6540	1294.6744	-15.71	0	36	0.28	1	U	K.LYFLQCNIPK.Y
☑	172	651.3455	1300.6764	1300.6921	-12.11	0	83	7.6e-006	1	U	R.QNISGLIQICR.D
☑	173	651.7880	1301.5614	1301.5744	-10.01	0	22	1.6	1	U	R.TECFTGSMTIR.I
	200	680.8173	1359.6200	1359.6347	-10.78	0	58	0.00087	1	U	K.YVYDPDFTGGVK.H
☑											
☑	239	721.3860	1440.7574	1440.7799	-15.62	0	43	0.053	1	U	R.VMYTLINSFVVR.L
☑	256	742.8691	1483.7236	1483.7381	-9.74	0	63	0.00049	1	U	R.ESFQNEFLMVLK.I
☑	263	750.8417	1499.6688	1499.6780	-6.09	0	78	5.9e-006	1	U	K.FASLNEYPETDSK.S
☑	266	753.3569	1504.6993	1504.6915	5.18	0	47	0.014	1	U	K.HMDFAALGICNTR.L
☑	292	793.8572	1585.6998	1585.7116	-7.46	0	55	0.00099	1	U	R.DVIVCCSEYSNIK.E
	381	955.4742	1908.9339	1907.9629	509	0	10	83	2	U	K.QFLLDILVYNENGENK.Y
☑	384	639.6462	1915.9167	1915.9428	-13.64	0	54	0.0032	1	U	R.IHEHDGTPYEHIVEIK.D
☑	392	979.4905	1956.9665	1956.9832	-8.53	0	69	0.00012	1	U	R.YENLVNWLSSYESLIK.T
☑	422	1028.0936	2054.1726	2054.1928	-9.82	0	0	3.4e+002	1	U	K.ILLWDVLNLPVLSLYQR.K
☑	435	704.9803	2111.9190	2111.9357	-7.94	0	53	0.0011	1	U	K.SEDDIELFLSGHYVDDMK.I
☑	443	1070.5364	2139.0582	2139.0789	-9.68	0	95	2.4e-007	1	U	K.LENFFNEWVYGSGVPILR.V
☑	444	714.0267	2139.0582	2139.0789	-9.68	0	(84)	3.3e-006	1	U	K.LENFFNEWVYGSGVPILR.V
☑	446	716.3739	2146.0999	2146.1157	-7.37	0	(20)	8.2	1	U	K.ISLEDANALSNYTPITPSIK.T
☑	447	1074.0572	2146.0999	2146.1157	-7.37	0	81	7.5e-006	1	U	K.ISLEDANALSNYTPITPSIK.T
☑	699	1055.4923	4217.9400	4217.9713	-7.41	0	33	0.067	1	U	R.CDQFIHDDPLQTNLNGLTSQNVLYSDNSIEQSHFLR.S

TAF14_YEAST **Mass:** 27423 **Score:** 605 **Matches:** 13(11) **Sequences:** 9(8) **emPAI:** 2.53
Transcription initiation factor TFIID subunit 14 OS=Saccharomyces cerevisiae (strain ATCC 204508 / S288c) GN=TAF14 PE=1 SV=1
☐ Check to include this hit in error tolerant search or archive report

	Query	Observed	Mr(expt)	Mr(calc)	ppm	Miss	Score	Expect	Rank	Unique	Peptide
☑	33	455.7332	909.4518	909.4596	-8.53	0	25	3.6	1	U	K.SLWDYVK.K
☑	282	777.3662	1552.7178	1552.7217	-2.51	0	106	1.6e-008	1	U	K.SGSTEETTANTGTIGK.R
☑	317	560.2915	1677.8527	1677.8739	-12.64	0	69	0.00015	1	U	K.VIYHLHPTFANPNR.T
☑	372	944.9662	1887.9178	1887.9248	-3.73	0	149	1.2e-012	1	U	K.LNEDDLVGVVQMVTDNK.T
☑	373	630.3138	1887.9197	1887.9248	-2.72	0	(74)	3.6e-005	1	U	K.LNEDDLVGVVQMVTDNK.T
☑	430	700.7105	2099.1097	2099.1164	-3.17	0	77	1.6e-005	1	U	K.TQQHILPEVPPVENFPVR.Q
☑	431	1050.5637	2099.1129	2099.1164	-1.64	0	(18)	12	1	U	K.TQQHILPEVPPVENFPVR.Q
☑	485	759.7246	2276.1521	2276.1729	-9.15	0	(50)	0.0084	1	U	R.IEEQGWGGFPLDISVFLLEK.A
☑	486	1139.0833	2276.1521	2276.1729	-9.14	0	55	0.0022	1	U	R.IEEQGWGGFPLDISVFLLEK.A
☑	572	887.1247	2658.3522	2658.3792	-10.17	1	60	0.00059	1	U	R.QWSIEIVLLDDEGKEIPATIFDK.V
☑	635	1055.5184	3163.5335	3163.5635	-9.48	0	88	5.8e-007	1	U	K.TPEMNVTNNVEEGEFIIDLYSLPEGLLK.S
☑	689	1000.2885	3997.1249	3997.1564	-7.89	1	(44)	0.0073	1	U	R.KIPHDLNFLQESYEVEHVIQIPLNKPLLTEELAK.S
☑	690	800.4322	3997.1249	3997.1564	-7.88	1	47	0.0034	1	U	R.KIPHDLNFLQESYEVEHVIQIPLNKPLLTEELAK.S

TAF6_YEAST **Mass:** 57980 **Score:** 550 **Matches:** 17(11) **Sequences:** 13(9) **emPAI:** 1.29
Transcription initiation factor TFIID subunit 6 OS=Saccharomyces cerevisiae (strain ATCC 204508 / S288c) GN=TAF6 PE=1 SV=1
☐ Check to include this hit in error tolerant search or archive report

	Query	Observed	Mr(expt)	Mr(calc)	ppm	Miss	Score	Expect	Rank	Unique	Peptide
☑	77	546.7649	1091.5153	1091.5347	-17.76	0	43	0.043	1	U	R.DVLTTDDVSK.A
☑	93	573.3041	1144.5936	1144.6088	-13.25	0	53	0.008	1	U	K.GVSVLEGESIR.F
☑	94	573.3052	1144.5958	1144.6088	-11.38	0	(32)	0.92	1	U	K.GVSVLEGESIR.F
	100	578.3486	1154.6827	1154.6910	-7.22	0	58	0.001	2	U	R.ILEIIEQAVK.F
☑	122	604.2895	1206.5645	1206.5743	-8.15	0	50	0.0087	1	U	R.VFGTYYGCLK.G
☑	136	622.8315	1243.6484	1243.6747	-21.20	0	32	0.9	1	U	R.CGVTIGFHILK.R
☑	193	676.3407	1350.6669	1350.6833	-12.11	0	42	0.069	1	U	R.FFLGNLNNWAR.L
☑	194	677.8587	1353.7028	1353.7180	-11.25	0	78	1.9e-005	1	U	R.DFAASLLDYVLK.K
☑	298	807.8664	1613.7183	1613.7321	-8.58	0	48	0.0063	1	U	K.DDSPQEIHEFLER.T
☑	401	662.0466	1983.1180	1983.1503	-16.31	0	(29)	0.58	1	U	K.EETQILVDTVISALLVLK.K
☑	402	992.5731	1983.1316	1983.1503	-9.44	0	92	2.7e-007	1	U	K.EETQILVDTVISALLVLK.K
☑	403	662.0516	1983.1329	1983.1503	-8.78	0	(72)	2.5e-005	1	U	K.EETQILVDTVISALLVLK.K
☑	423	1030.0014	2057.9883	2057.9946	-3.06	0	75	2.1e-005	1	U	R.VLNVEPLYGYYDGSEVNK.A
☑	606	948.1187	2841.3342	2841.3417	-2.64	0	132	2.5e-011	1	U	R.LVFNESGITLDNIEEHLNDDSNPTR.T
☑	607	711.3415	2841.3370	2841.3417	-1.64	0	(67)	7.6e-005	1	U	R.LVFNESGITLDNIEEHLNDDSNPTR.T
☑	634	790.1714	3156.6565	3156.6720	-4.90	0	21	2.7	1	U	R.LPTFTTHWLAVEGVQPAIIQNPNLNDIR.V
☑	717	1010.3225	5046.5759	5046.6110	-6.95	0	32	0.065	1	U	R.GAIVTALNDNSLQTPVTSTTASASVTDTGASQHLSNVKPGQNTEVKPLVK.

TAF1_YEAST **Mass:** 120849 **Score:** 537 **Matches:** 15(11) **Sequences:** 13(10) **emPAI:** 0.45
Transcription initiation factor TFIID subunit 1 OS=Saccharomyces cerevisiae (strain ATCC 204508 / S288c) GN=TAF1 PE=1 SV=1
☐ Check to include this hit in error tolerant search or archive report

	Query	Observed	Mr(expt)	Mr(calc)	ppm	Miss	Score	Expect	Rank	Unique	Peptide
☑	80	551.2828	1100.5511	1100.5713	-18.37	0	56	0.003	1	U	K.INNLEELEK.Q
☑	163	642.3735	1282.7324	1282.7496	-13.37	0	39	0.099	1	U	K.LLQLELANLEK.S
☑	166	645.8135	1289.6125	1289.6324	-15.42	0	37	0.19	1	U	K.NNLLQSNSSASR.R
☑	171	433.8928	1298.6567	1298.6942	-28.90	1	32	0.67	1	U	R.KANEQDTLRPK.L
☑	190	673.8454	1345.6763	1345.6878	-8.53	0	46	0.037	1	U	K.HDISGTDFLLTK.S
☑	259	746.4012	1490.7878	1490.8093	-14.40	0	71	9.9e-005	1	U	K.LPVGETHVLGVQDK.S
☑	260	497.9366	1490.7879	1490.8093	-14.39	0	(40)	0.11	1	U	K.LPVGETHVLGVQDK.S
☑	283	778.9121	1555.8097	1555.8246	-9.58	0	58	0.0017	1	U	K.SLEENLLPWNITK.N
☑	336	579.6605	1735.9598	1735.9985	-22.26	1	41	0.054	1	U	R.RGLIHVSIDELFPIK.E
☑	342	889.8990	1777.7835	1777.7966	-7.38	0	123	1.7e-010	1	U	R.TENSVDNGSDLAGVTDGK.A
☑	405	996.4696	1990.9246	1990.9371	-6.30	0	83	3.1e-006	1	U	K.TISEDLLIATDDWDQEK.I
☑	490	765.7499	2294.2278	2294.2481	-8.87	0	(66)	0.00017	1	U	K.QQLDSSNLILPLNETILQQK.F
☑	491	1148.1212	2294.2278	2294.2481	-8.87	0	92	4.3e-007	1	U	K.QQLDSSNLILPLNETILQQK.F
☑	498	774.3663	2320.0770	2320.0981	-9.08	0	78	7.1e-006	1	U	R.AMIQIHGVGDPTGCGEGFSFLK.T
☑	555	856.0997	2565.2774	2565.2976	-7.86	0	87	1.1e-006	1	U	R.NINHLFTVGQTFPVEEIPGPNSR.K

TAF3_YEAST **Mass:** 40385 **Score:** 444 **Matches:** 13(9) **Sequences:** 13(9) **emPAI:** 1.03
Transcription initiation factor TFIID subunit 3 OS=Saccharomyces cerevisiae (strain ATCC 204508 / S288c) GN=TAF3 PE=1 SV=1
☐ Check to include this hit in error tolerant search or archive report

	Query	Observed	Mr(expt)	Mr(calc)	ppm	Miss	Score	Expect	Rank	Unique	Peptide
☑	34	464.3041	926.5937	926.6164	-24.54	0	43	0.021	1	U	R.ISILQLLK.A
☑	199	454.2053	1359.5941	1359.6280	-24.94	0	51	0.0023	1	U	R.HHHTTITNEDR.M
☑	250	737.8634	1473.7123	1473.7252	-8.78	0	63	0.00047	1	U	M.TTNNDFYFALLR.I
☑	313	835.9597	1669.9049	1669.9138	-5.32	0	86	2.7e-006	1	U	R.ITALPTVELLQSEEK.E
☑	316	839.9336	1677.8527	1677.8760	-13.86	0	2	6.8e+002	1	U	R.ARPSLVDVMTDLYAK.F
☑	322	846.4618	1690.9089	1690.9254	-9.70	0	109	1.2e-008	1	U	K.FLSLLASEVSSIAQAR.C
☑	387	968.4576	1934.9007	1934.9109	-5.27	0	77	1.2e-005	1	U	R.DEDALTEIQENPFVTSK.L
☑	436	1058.0212	2114.0278	2114.0531	-11.97	0	55	0.0026	1	U	K.TELIEDLINNNGLDDWIK.L
☑	453	1079.5466	2157.0786	2157.0953	-7.76	0	9	1e+002	1	U	K.ESDPLSAIPDYLNQLLQNK.G
☑	478	748.3780	2242.1120	2242.1481	-16.09	1	10	75	1	U	R.KTELIEDLINNNGLDDWIK.L
☑	602	940.4421	2818.3045	2818.3396	-12.44	0	75	9.8e-006	1	U	R.LENITLSFEDEELESLGEVEGPNQK.S
☑	676	953.2489	3808.9664	3808.9985	-8.44	1	15	7.4	1	U	R.ITALPTVELLQSEEKESDPLSAIPDYLNQLLQNK.G
☑	707	1141.7995	4563.1689	4563.2174	-10.62	0	100	1.2e-008	1	U	R.CDQDDTIALQDITLALENLGIVKPTNVLDVYDENSELSSSR.G

TAF12_YEAST **Mass:** 61150 **Score:** 425 **Matches:** 14(10) **Sequences:** 14(10) **emPAI:** 0.69
Transcription initiation factor TFIID subunit 12 OS=Saccharomyces cerevisiae (strain ATCC 204508 / S288c) GN=TAF12 PE=1 SV=1
☐ Check to include this hit in error tolerant search or archive report

	Query	Observed	Mr(expt)	Mr(calc)	ppm	Miss	Score	Expect	Rank	Unique	Peptide
☑	28	437.7330	873.4514	873.4708	-22.29	0	52	0.012	1	U	K.TPAPVAYR.S
☑	76	545.7853	1089.5561	1089.5720	-14.53	0	30	1.5	1	U	K.LWQNFQVR.H
☑	84	560.7793	1119.5441	1119.5560	-10.65	0	61	0.00086	1	U	R.IPGYSADEIR.S
☑	114	596.8126	1191.6107	1191.6248	-11.84	0	68	0.00022	1	U	K.QVYDALTLNR.R
	137	623.8084	1245.6023	1244.6109	797	0	12	67	2	U	K.NNGNNVASLNTK.K
☑	138	625.2900	1248.5654	1248.5809	-12.34	0	53	0.0028	1	U	K.LPPYEMDTQR.V
☑	147	632.8101	1263.6056	1263.6208	-11.96	0	71	7.6e-005	1	U	K.FLQESTQQQR.V
☑	220	703.8354	1405.6562	1405.6738	-12.51	1	13	39	1	U	R.KWNPSQNYNQK.L
☑	295	803.8773	1605.7401	1605.7522	-7.52	0	64	0.00024	1	U	K.QVLTPEENQEYEK.L
☑	301	814.4290	1626.8434	1626.8464	-1.89	0	5	3.3e+002	1	U	K.QTEPAIPISENISTK.T
☑	356	914.9431	1827.8717	1827.8823	-5.82	0	92	4.7e-007	1	U	K.SQQQQNTITATNNPR.G
☑	379	951.9817	1901.9488	1901.9582	-4.94	0	70	0.0001	1	U	K.ISSSNSTEIPSVTGPDALK.S
☑	513	787.7267	2360.1582	2360.1754	-7.28	0	98	1e-007	1	U	R.SNRPTITGGSAMNASALNTPATTK.L
☑	695	1025.7227	4098.8617	4098.9104	-11.87	0	59	0.00016	1	U	K.TVGIDEGDGETVIDGDVEELLLDLADDFVTNVTAFSCR.L

TAF10_YEAST **Mass:** 23005 **Score:** 369 **Matches:** 6(4) **Sequences:** 5(3) **emPAI:** 0.97
Transcription initiation factor TFIID subunit 10 OS=Saccharomyces cerevisiae (strain ATCC 204508 / S288c) GN=TAF10 PE=1 SV=1
☐ Check to include this hit in error tolerant search or archive report

	Query	Observed	Mr(expt)	Mr(calc)	ppm	Miss	Score	Expect	Rank	Unique	Peptide
☑	51	496.7347	991.4548	990.4883	976	0	34	0.27	1	U	K.NGFNVADVR.V
☑	419	1026.9763	2051.9380	2051.9436	-2.75	0	111	3.3e-009	1	U	R.EAVVDDGSENAFGIPEFTR.K
☑	601	934.1436	2799.4090	2799.4012	2.77	0	107	1.1e-008	1	U	R.QLLQGQQQPGVQQISQQQHQQNEK.T
☑	610	956.8307	2867.4702	2867.4817	-4.04	0	118	9.4e-010	1	U	K.VVLTVNDLSSAVAEYGLNIGRPDFYR.-
☑	611	717.8759	2867.4747	2867.4817	-2.46	0	(102)	3.1e-008	1	U	K.VVLTVNDLSSAVAEYGLNIGRPDFYR.-
☑	655	1118.5522	3352.6348	3352.6710	-10.78	1	0	3.2e+002	1	U	K.DKTLEEILEMMDSTPPIIPDAVIDYYLTK.N

TAF4_YEAS7 **Mass:** 42338 **Score:** 328 **Matches:** 10(8) **Sequences:** 10(8) **emPAI:** 0.83
Transcription initiation factor TFIID subunit 4 OS=Saccharomyces cerevisiae (strain YJM789) GN=TAF4 PE=3 SV=1
☐ Check to include this hit in error tolerant search or archive report

	Query	Observed	Mr(expt)	Mr(calc)	ppm	Miss	Score	Expect	Rank	Unique	Peptide
☑	29	444.7412	887.4679	887.4825	-16.41	0	20	21	1	U	R.ASNVTAGLR.A
☑	49	494.7684	987.5223	987.5461	-24.10	1	56	0.0045	1	U	R.RSEVSAALR.A
☑	78	550.2655	1098.5164	1098.5305	-12.84	0	65	0.00023	1	U	K.IDSEETLHR.A
☑	113	596.2965	1190.5785	1190.5931	-12.31	0	52	0.0076	1	U	K.EQNFNLTPTK.N
☑	127	607.8511	1213.6877	1213.7030	-12.65	0	65	0.00035	1	U	R.DILTNAIVISR.H
☑	265	752.8698	1503.7250	1503.7416	-11.01	0	84	3.5e-006	1	U	R.EEEALLNSSINASK.S
☑	290	790.9084	1579.8023	1579.8206	-11.55	0	59	0.0015	1	U	K.ASELSHSLPSPSQIK.S
☑	388	646.3383	1935.9930	1936.0054	-6.42	0	90	8.4e-007	1	U	K.QYGWLTSSVNKPTSLGAK.S
☑	469	731.3647	2191.0723	2191.0997	-12.48	0	9	90	1	U	K.IPNHLPFLHPEQVSNYMR.K
☑	499	775.3592	2323.0558	2323.0677	-5.12	0	80	3.7e-006	1	U	K.STAHVSSTHNDAAGNTDDSVLPK.N

TAF9_YEAST **Mass:** 17361 **Score:** 312 **Matches:** 6(5) **Sequences:** 5(4) **emPAI:** 1.44
Transcription initiation factor TFIID subunit 9 OS=Saccharomyces cerevisiae (strain ATCC 204508 / S288c) GN=TAF9 PE=1 SV=1
☐ Check to include this hit in error tolerant search or archive report

	Query	Observed	Mr(expt)	Mr(calc)	ppm	Miss	Score	Expect	Rank	Unique	Peptide
	20	404.7233	807.4321	807.4491	-21.01	0	1	1.3e+003	7	U	R.YTQGVLK.D
☑	181	657.8446	1313.6746	1313.6914	-12.78	0	66	0.00035	1	U	K.ALPQVMGTWGVR.L
☑	427	1037.9741	2073.9337	2073.9451	-5.50	0	111	2.9e-009	1	U	K.NSVGSVSEVGPDSTQEETPR.D
☑	539	834.0529	2499.1368	2499.1514	-5.82	0	123	1.7e-010	1	U	K.DALVYNDYAGSGNSAGSGLGVEDIR.L
☑	650	829.6808	3314.6939	3314.7234	-8.88	0	(52)	0.0024	1	U	R.LLHLLLASQSIHQYEDQVPLQLMDFAHR.Y
☑	651	663.9461	3314.6940	3314.7234	-8.87	0	59	0.00051	1	U	R.LLHLLLASQSIHQYEDQVPLQLMDFAHR.Y

TAF7_YEAST **Mass:** 67571 **Score:** 263 **Matches:** 7(7) **Sequences:** 7(7) **emPAI:** 0.39
Transcription initiation factor TFIID subunit 7 OS=Saccharomyces cerevisiae (strain ATCC 204508 / S288c) GN=TAF7 PE=1 SV=1
☐ Check to include this hit in error tolerant search or archive report

	Query	Observed	Mr(expt)	Mr(calc)	ppm	Miss	Score	Expect	Rank	Unique	Peptide
☑	140	626.8319	1251.6491	1251.6724	-18.57	0	45	0.041	1	U	K.HGISPPLYNVR.N
☑	141	627.8289	1253.6432	1253.6615	-14.66	0	64	0.00045	1	U	K.EEIHTIASAVGK.Q
☑	175	652.3203	1302.6261	1302.6416	-11.87	0	60	0.001	1	U	K.QLQQTEDSVQK.Q
☑	182	657.8786	1313.7427	1313.7595	-12.76	0	59	0.0013	1	U	R.ILPDIQLEFVK.N
☑	211	698.3285	1394.6424	1394.6565	-10.14	0	69	8.9e-005	1	U	K.QAEEVSYDLVDK.S
☑	248	735.3540	1468.6933	1468.7045	-7.61	0	94	3.2e-007	1	U	K.NSLESGDYSGISIK.W
☑	522	802.7369	2405.1889	2405.2074	-7.68	0	72	4.9e-005	1	U	R.QHTELLADELNELETTLAHTK.H

TAF8_YEAST **Mass:** 58007 **Score:** 256 **Matches:** 5(5) **Sequences:** 5(5) **emPAI:** 0.32
Transcription initiation factor TFIID subunit 8 OS=Saccharomyces cerevisiae (strain ATCC 204508 / S288c) GN=TAF8 PE=1 SV=1

☐ Check to include this hit in error tolerant search or archive report

	Query	Observed	Mr(expt)	Mr(calc)	ppm	Miss	Score	Expect	Rank	Unique	Peptide
☑	132	611.3470	1220.6794	1220.6989	-16.02	0	47	0.021	1	U	R.NLHNLTLLQR.R
☑	312	832.9198	1663.8250	1663.8377	-7.63	0	128	1.7e-010	1	U	K.TSESGTGTQSTIVQLR.N
☑	516	1188.0700	2374.1254	2374.1481	-9.57	0	46	0.014	1	U	R.EFNLDAPSLYQQFQASEFIK.S
☑	528	820.1125	2457.3156	2457.3366	-8.55	0	67	0.0001	1	U	R.NLPDLTEISHLEIDAPVVEILK.K
☑	694	1012.4754	4045.8723	4045.9141	-10.31	0	52	0.00098	1	U	K.SLSHISSASNTPQPPGLDDEDAIEQQLEIWGNALEER.K

TAF11_YEAST **Mass:** 40656 **Score:** 167 **Matches:** 7(4) **Sequences:** 7(4) **emPAI:** 0.48
Transcription initiation factor TFIID subunit 11 OS=Saccharomyces cerevisiae (strain ATCC 204508 / S288c) GN=TAF11 PE=1 SV=1
☐ Check to include this hit in error tolerant search or archive report

	Query	Observed	Mr(expt)	Mr(calc)	ppm	Miss	Score	Expect	Rank	Unique	Peptide
	116	599.2755	1196.5365	1196.5244	10.1	0	12	31	3	U	R.TQGEGQGSMFR.-
☑	137	623.8084	1245.6023	1245.6313	-23.26	0	63	0.00057	1	U	R.TGGTSINNQLNK.Y
☑	167	645.8388	1289.6630	1289.6827	-15.27	0	47	0.03	1	U	R.ETTLGNSLLQSK.S
☑	242	726.8643	1451.7140	1451.7521	-26.22	0	10	1e+002	1	U	K.YNNSPLLPEHIR.E
☑	275	765.4046	1528.7947	1528.8209	-17.16	1	53	0.0066	1	U	K.LLVTNLDKDQTNR.F
☑	299	808.9257	1615.8368	1615.8529	-9.98	0	84	4.3e-006	1	U	K.LASTVANQTISENIR.V
☑	363	920.9851	1839.9556	1839.9771	-11.64	0	34	0.36	1	U	K.VNYPPILTIANYFSTK.Q

TAF13_YEAST **Mass:** 19235 **Score:** 38 **Matches:** 1(1) **Sequences:** 1(1) **emPAI:** 0.18
Transcription initiation factor TFIID subunit 13 OS=Saccharomyces cerevisiae (strain ATCC 204508 / S288c) GN=TAF13 PE=1 SV=2
☐ Check to include this hit in error tolerant search or archive report

	Query	Observed	Mr(expt)	Mr(calc)	ppm	Miss	Score	Expect	Rank	Unique	Peptide
☑	719	1049.2987	5241.4572	5241.5023	-8.62	0	38	0.01	1	U	K.DVSSLLYAYGDVPQPLQATVQCLDELVSGYLVDVCTNAFHTAQNSQR.N

Supplementary Figure S2. Mass spectrometry results showing that all 14 TAFs are present in the purified complex.

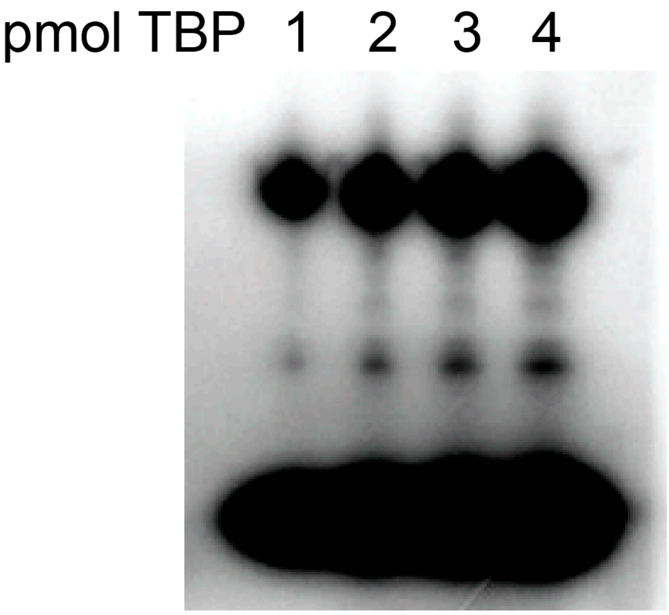

Supplementary Figure S3. EMSA in 2% agarose gel for TBP on *RPS5*. TBP alone binds *RPS5* forming at least two specific complexes with one or more TBP molecules bound.

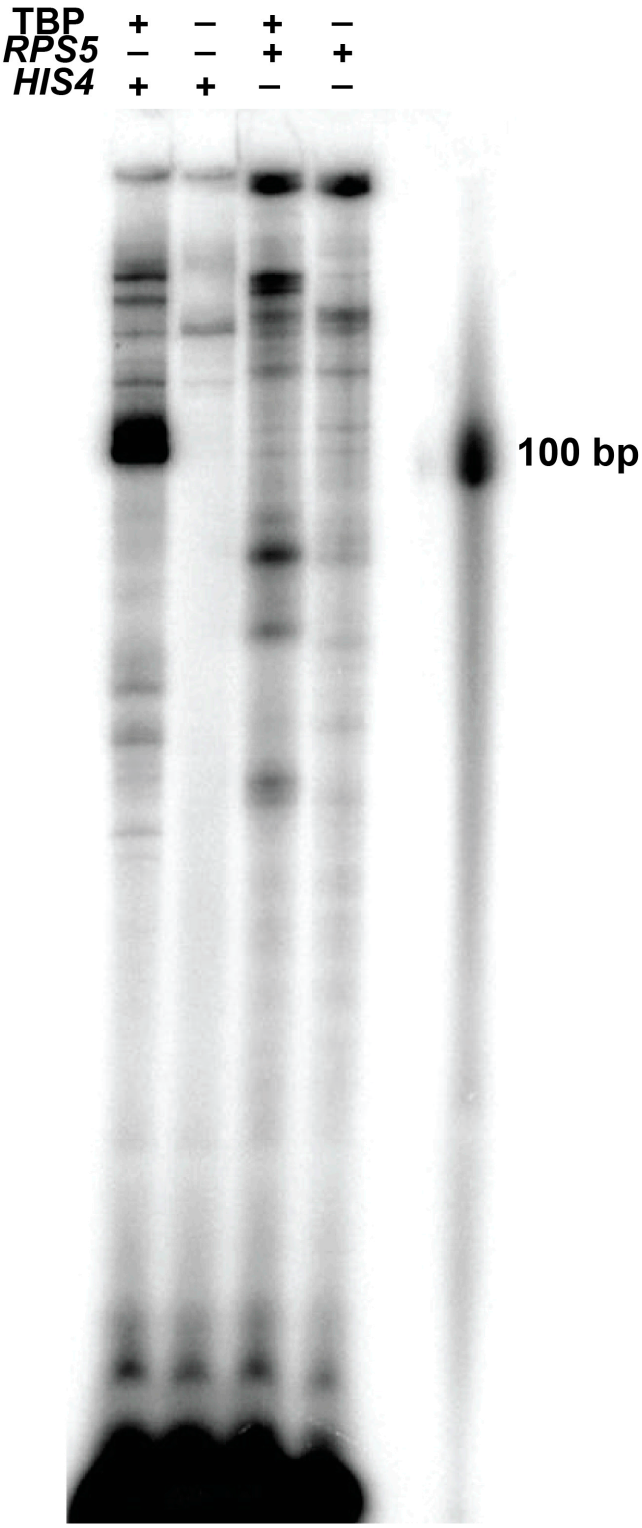

Supplementary Figure S4. Positive and negative controls for transcription run-off assays. Lane 1: Run-off products from the *HIS4* TATA promoter to assess the activity of purified GTFs without the TAF complex. Lane 2: Negative control; same as Lane 1 minus TBP and TAFs. Lane 3: Run-off products from the *RPS5* TATA-less promoter in the presence of TBP only (minus TAFs). This establishes the TBP-directed transcription background. Lane 4: Negative control to establish *RPS5* background transcription (minus TBP minus TAFs). Comparing Lanes 3 and 4 reveals the strongest top band that is much longer than 100 bp, inconsistent with the mRNA products detected in vivo. This band corresponds to an artificial

transcript from a TSS located approximately 25 bp downstream of the pseudo-TATA box. Rightmost lane: 100 bp ladder.

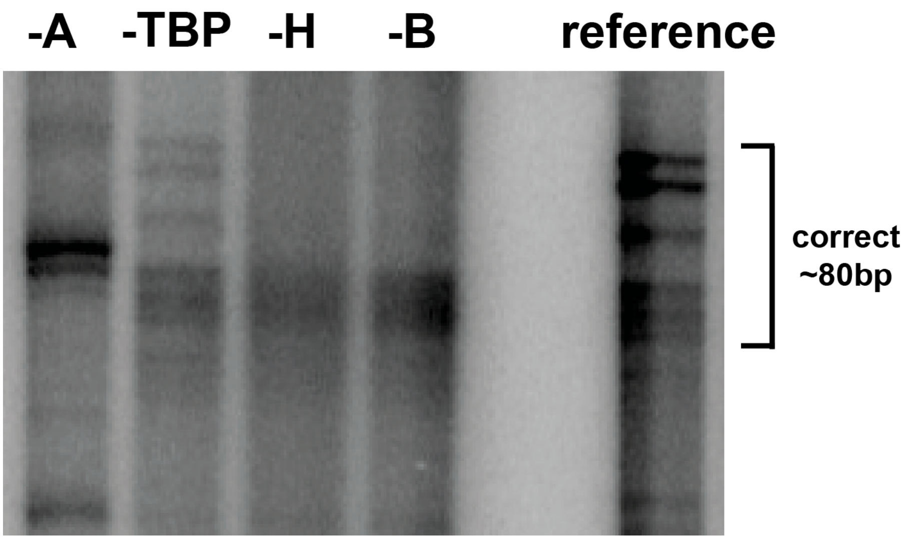

Supplementary Figure S5. Negative controls for transcription run-off assays on *RPS5* with TAFs present. Lane 1: Omitting TFIIA. Lane 2: Omitting TBP. Lane 3: Omitting TFIIH. Lane 4: Omitting TFIIB. Faint correct bands are observed when TBP is omitted, which we attribute to the 3% contamination of TBP in our TAF complex preparation.
